# Novel orbivirus in *Amblyomma tholloni* ticks parasitizing African savanna elephants (*Loxodonta africana*) in Zambia

**DOI:** 10.1007/s11262-025-02187-7

**Published:** 2025-09-22

**Authors:** Daniella E. Chusyd, Lisa Olivier, Moses Kasongo, Webster Mwaanga, Tony L. Goldberg

**Affiliations:** 1 Department of Environmental and Occupational Health, Indiana University-Bloomington, Bloomington, IN USA; 2 Game Rangers International, Lusaka, Zambia; 3 Department of Pathobiological Sciences, School of Veterinary Medicine, University of Wisconsin-Madison, Madison, WI USA

**Keywords:** Tick-borne virus, Viral discovery, Arbovirus, Kafue National Park

## Abstract

Orbiviruses are emerging pathogens of public and veterinary health concern. This study reports the discovery and genomic characterization of a novel orbivirus in adult ticks collected from African savanna elephants (*Loxodonta africana*) in Kafue National Park, Zambia. Six ticks were identified through genetic analyses; one *Rhipicephalus maculatus* and five *Amblyomma tholloni*. Salivary gland and blood meal samples were dissected for virus characterization. Total nucleic acids were extracted and reverse-transcribed, and libraries were prepared for deep sequencing. De novo assembly of trimmed sequences recovered four novel viruses designated “zaloxo” to indicate their association with **Za**mbian elephants (genus ***Loxodonta***), abbreviated ZXLV-1 to ZXLV-4. ZXLV-1, a member of the genus *Orbivirus*, belongs to the same subclade of dsRNA orbiviruses as bluetongue virus, epizootic hemorrhagic disease virus, and African horse sickness virus. ZXLV-2 and ZXLV-3 are most closely related to dsRNA *Totiviridae* and ssDNA *Circoviridae* viruses, respectively**.** ZXLV-4 belongs to the genus *Alphapolyomavirus*, dsDNA viruses which are ubiquitous in mammals but have unclear associations with disease. While the pathogenicity of these viruses remains unclear, these results suggest that a hidden diversity of viruses likely exists in elephant ectoparasites, warranting attention from the perspective of both health and conservation.

**Supplementary Information:**

The online version contains supplementary material available at 10.1007/s11262-025-02187-7.

In recent decades, numerous viruses belonging to the genus *Orbivirus* (family *Sedoreoviridae*) have been described in both arthropod vectors and vertebrate hosts. Orbiviruses are typically transmitted by mosquitoes, sandflies, biting midges, and ticks [1–3]. The genome consists of 10 linear segments of double-stranded RNA, and the virion consists of an icosahedral, triple-layered capsid without an outer envelope [4]. Several members of the *Orbivirus* genus, including bluetongue virus (BTV), epizootic (EHDV), and African horse sickness virus (AHSV), can cause severe illnesses in both wild and domestic animals [5]. BTV primarily affects sheep, leading to a high mortality, but it can also cause disease in other ungulates [6, 7]. EHDV causes hemorrhagic disease in deer and subclinical infections in domestic ruminants [8]. AHSV can infect all equine species, causing respiratory and circulatory disease, which can be fatal [9].

Here we report the discovery and genomic characterization of a novel orbivirus in adult ticks collected from African savanna elephants (*Loxodonta africana*) in Kafue National Park, Zambia. In July 2021, ticks (*n* = 6, four of which were engorged) were removed directly from orphaned elephants (*n* = 5) living under human care but residing in the national park. After collection, ticks were submerged in RNAlater nucleic acid preservation solution (Qiagen, Hilden, Germany) and stored at -20 °C until shipment to the USA. Ethical approvals for the study and shipment of ticks were obtained from the Indiana University Animal Use and Care Committee (22–022), Game Rangers International, Zambia’s Department of National Parks and Wildlife (DNPW; NPW/8/27/1), and the US Center for Disease Control and Prevention (20,211,018-3861A).

Ticks were processed using methods previously described for other hematophagous arthropods [10–13]. Ticks were first surface-sterilized using dilute bleach followed by three rinses with nuclease-free water to remove external microbes [14]. Each tick was dissected using sterile instruments, with precautions taken not to cross-contaminate internal structures. Salivary glands were removed and washed three times in 1 ml of molecular grade Hank’s balanced salt solution (HBSS). For the four engorged ticks, approximately 10 mg of solidified blood meal was removed from the midgut and processed separately. Salivary gland and blood meal samples were homogenized in HBSS in PowerBead tubes with 2.38 mm metal beads (Qiagen, Hilden, Germany) using four 30 s cycles in an orbital homogenizer (Minibeadbeater, BioSpec Products, Bartlesville OK, USA).

For genetic identification of ticks, DNA was extracted from 50 µl of tick salivary gland homogenate using the DNeasy Blood & Tissue Kit (Qiagen). A segment of the cytochrome oxidase subunit 1 (cox1) gene was amplified by polymerase chain reaction (PCR) with barcoding primers LCO1490 (5′-GGTCAACAAATCATAAAGATATTGG-3′) and HC02198 (5′-TAAACTTCAGGGTGACCAAAAAATCA-3′) [15] using the HotStarTaq system (Qiagen). Amplicons were elecrophoresed on 1% agarose gels stained with ethidium bromide and visualized under ultraviolet light. Amplicons were then Sanger sequenced on ABI 3730xl sequencers (Thermo Fisher Scientific, Waltham, MA, USA) at a commercial facility (Functional Biosciences, Madison, WI, USA). Resulting 658 base pair sequences (GenBank PV185184-PV185189) identified one tick as *Rhipicephalus maculatus* (98.9% identical to GenBank KY457540.1) and five ticks as *Amblyomma tholloni* (99.7 – 99.9% identical to GenBank KT307493.1) (Fig. 1S).

**Fig. 1:**
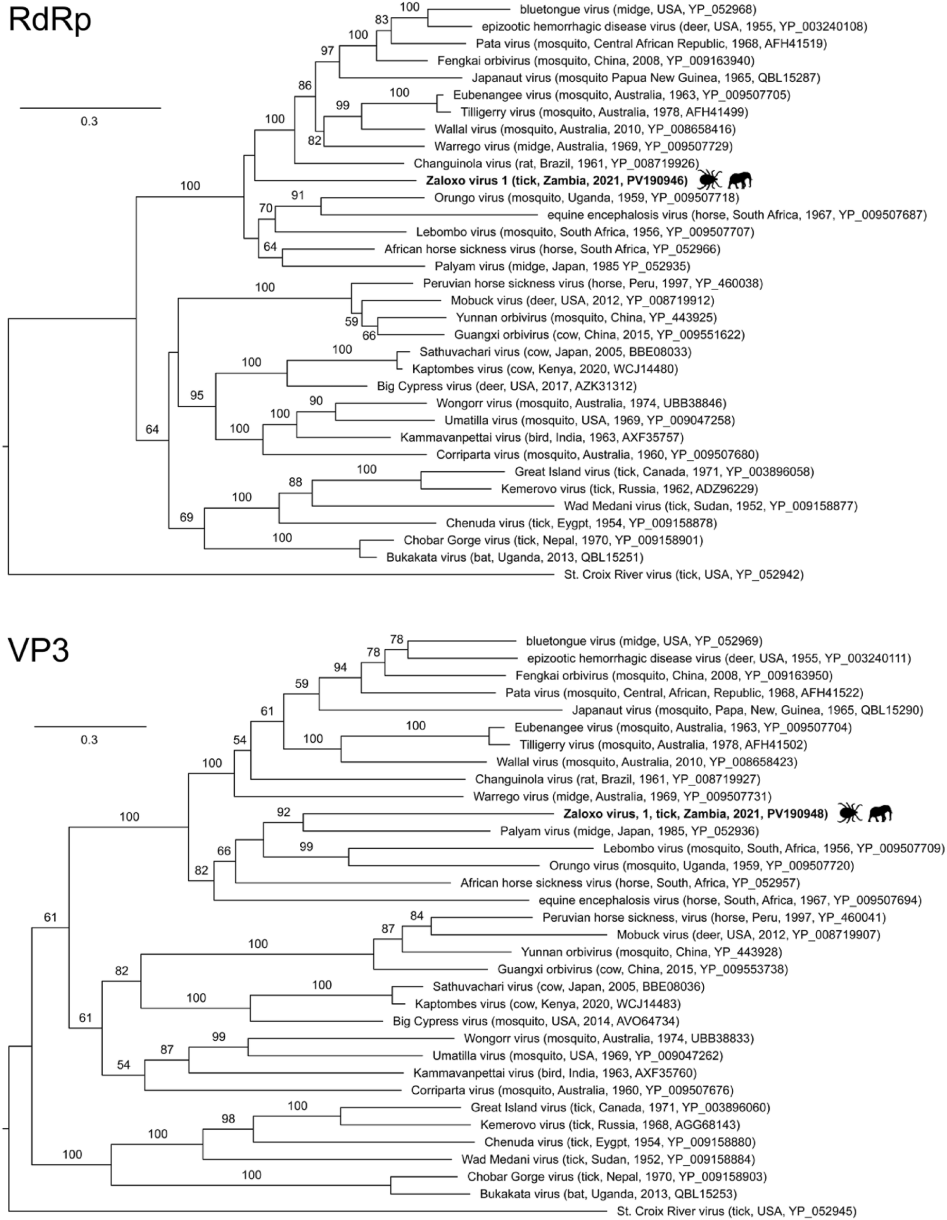
Maximum likelihood phylogenetic trees of orbiviruses based on amino acid (aa) sequences of RNA-dependent RNA polymerase (VP1; 558 aa alignment) and Inner capsid shell protein (VP3; 611 aa alignment). Taxon names are followed in parentheses by (where available) host, country, year, and GenBank accession number. The RdRp and T2 trees were inferred from 558- to 611-position cleaned alignments, respectively. Newly generated sequences from an *Amblyomma tholloni* tick parasitizing an African savanna elephant in Zambia are shown in bold, with silhouettes. Numbers beside branches indicate bootstrap values based on 1,000 replicates (values ≤ 50% are not shown). Scale bars indicate amino acid substitutions per site

For virus characterization, 250 µl of sample homogenate (salivary gland or blood meal) was centrifuged and lightly digested with nucleases to reduce non-encapsulated genetic material, as previously described [11–13]. Total nucleic acids were then extracted from all samples, RNA was reverse transcribed to cDNA using the SuperScript IV System (Thermo Fisher, Waltham, MA, USA), and libraries were prepared using the Nextera XT DNA sample preparation kit (Illumina, San Diego, CA, USA) and sequenced on a MiSeq instrument (V3 chemistry, 600 cycle paired-end; Illumina), also as previously described [11–13]. Resulting sequences were trimmed to a quality (Phred) score of ≥ Q30 and length ≥ 50 using CLC Genomics Workbench, and reads of known contaminants, ribosomal sequences, the assembled *Rhipicephalus microplus* genome (GenBank GCA_013339725.1) and the assembled African elephant genome (GenBank GCA_030014295.1) were subtracted in silico. Remaining sequences underwent de novo assembly using metaSPAdes v.3.15.5 [16]. Resulting contigs ≥ 500 nt were compared to the NCBI non-redundant (nr) protein database using 6-frame translation, implemented with DIAMOND [17]. Putative viral hits were then examined manually using ORF finder [18] to verify deduced open reading frames and blastn and blastp [19] to verify the results from DIAMOND against the full GenBank databases. Bacteriophage sequences were not considered further, because the goal of the study was to identify viruses capable of infecting eukaryotes.

Four novel viruses were identified, which were named using the term “zaloxo” to indicate their association with **Za**mbian elephants (genus ***Loxodonta***) (Table 1S). ZXLV-1 is a member of the genus *Orbivirus*, family *Sedoreoviridae*, and is distinct from any of its congenerics (Fig. 1). ZXLV-1 is, however, part of the same subclade of orbiviruses as BTV, EHDV and AHSV, based on phylogenies inferred from both RdRp and VP3 inferred amino acid sequences (Fig. 1). ZXLV-1 has 10 segments encoding canonical orbivirus proteins [1], ranging from 59 to 21% identical at the amino acid level to BTV, an exemplar of the genus (Table 1). BTV, EHDV, and AHSV all have genomes of approximately 19,200 nt arranged in 10 segments approximately 3.90 kb–0.80 kb nt in length [20–22]. The total length of the ZXLV-1 genome sequenced in the present study was 12,453 nt, which, assuming that ZXLV-1 has a similar total genome size, represents approximately 65% of the genome. ZXLV-1 was present in the salivary gland and blood meal of a single *Amblyomma tholloni* tick (Table 1S), suggesting that it could be vector-borne. This discovery is particularly significant given the historical context of orbiviruses, and associated eradication challenges, in Southern Africa [23–26]. Orbiviruses are primarily transmitted by arthropod vectors, such that climate change and its effects on vector distribution could impact their transmission in the future [27].

**Table 1:** Comparison of ZLXV-1, a novel orbivirus from an elephant tick in Zambia, to bluetongue virus

Segment	Accession	Contig length (nt)^1^	Coverage^1^	Protein^1^	Product^1^	% id (AA)^2^	Accession (ref)^2^
1	PV190946	1980	14.74	VP1	RNA-dependent RNA polymerase	57.38	YP_052968.1
2	PV190947	771	9.89	VP2	Serotype-specific capsid protein	21.24	YP_052958.1
3	PV190948	2478	22.75	VP3	Inner capsid shell protein	59.25	YP_052959.1
4	PV190949	1836	19.79	VP4	Core protein	50.16	YP_052969.2
5	PV190950	1482	29.49	NS1	Hydrophobic tubular protein	24.49	YP_052970.1
6	PV190951	1392	25.50	VP5	Inner layer of outer capsid protein	42.32	YP_052955.1
7	PV190952	567	6.04	VP7	Inner capsid protein	33.54	YP_052967.1
8	PV190953	768	33.02	NS2	Phosphorylated matrix protein	31.08	YP_052952.1
9	PV190954	918	16.85	VP6	Helicase/NTPase	46.03	YP_052953.2
10	PV190955	261	3.08	NS3	Membrane glycoprotein	40.91	YP_052960.1

^1^Length and average coverage of the contiguous sequence (contig), viral protein encoded, and protein product description; and ^2^Percent amino acid identity to bluetongue virus (BTV, the *Orbivirus* exemplar), and accession number of BTV reference sequence

ZXLV-2 and ZXLV-3 are most closely related to dsRNA and ssDNA viruses of the *Totiviridae* and *Circoviridae* families, respectively, the former of which was identified in a tick and the latter in an unspecified source. These viruses were present in two bloodmeal samples and one salivary gland sample (Table 1S). Because of the families to which ZXLV-2 and ZXLV-3 belong, they are likely of environmental or invertebrate origin. ZXLV-4 is in the genus *Alphapolyomavirus*, family *Polyomaviridae*, which are ubiquitous dsDNA viruses in mammals but have unclear associations with disease in most cases [28]. ZXLV-4 was present in most salivary gland and blood meal samples, suggesting that it could be vector-borne.

It is unclear whether ZXLV-1 causes disease. The elephant from which the ZXLV-1-positive tick was sampled appeared clinically healthy at the time of sampling and had no unusual clinical history. Similarly, the clinical implications of the other viruses identified are unclear, although the pathogenic potential of ZXLV-4 is probably higher than ZXLV-2 or ZXLV-3 (although it is probably low overall). Additional work would be required to assess the pathogenicity of ZXLV-1 and the other viruses identified in this study. However, African elephants are endangered and, for this and many other reasons, cannot be experimentally infected. Therefore, the most appropriate way to assess any health effects of these viruses would be regular testing of blood for these viruses and simultaneous clinical monitoring of the elephants themselves. Should any of these viruses be associated with disease, tick control would be a logical preventive strategy.

## Supplementary Information

Below is the link to the electronic supplementary material.

Supplementary file1 (DOCX 332 KB)

## Data Availability

Mitochondrial COX1 sequences were deposited in GenBank under accession numbers PV185184-PV185189. Virus sequences were deposited in GenBank under accession numbers PV190946-PV190955.
