# Supplementary material for: Novel orbivirus in Amblyomma tholloni ticks parasitizing African savanna elephants (Loxodonta africana) in Zambia
Source: Virus Genes. 2025 Sep 22;61(6):742–6. doi: 10.1007/s11262-025-02187-7 (PMC12678566; doi:10.1007/s11262-025-02187-7)
Supplement: Supplementary file 1 — Supplementary file1 (DOCX 332 KB) [file 11262_2025_2187_MOESM1_ESM.docx]

Supplemental File

**Novel orbivirus in ticks (*Amblyomma tholloni*) parasitizing African savanna elephants (*Loxodonta* africana) in Zambia**

Daniella E. Chusyd^1*^, Lisa Olivier^2^, Moses Kasongo^2^, Webster Mwaanga^2^, Tony Goldberg^3*^

^1^Department of Environmental and Occupational Health, Indiana University-Bloomington, Bloomington, IN, USA

^2^Game Rangers International, Lusaka, Zambia

^3^Department of Pathobiological Sciences, School of Veterinary Medicine, University of Wisconsin, Madison, WI, USA

**Table 1S.** Viruses in ticks parasitizing African savanna elephants in Kafue National Park, Zambia.

| **Virus** | **Abbrev** | **Contig Length (nt)** | **Coverage^1^** | **Accession^2^** | **Closest match (host, location, year, accession)^3^** | **Genome^3^** | **Family^3^** | **Genus^3^** | **E-value^3^** | **% ID (aa) ^3^** | **SG^3^** | **BM^4^** |
| --- | --- | --- | --- | --- | --- | --- | --- | --- | --- | --- | --- | --- |
| Zaloxo virus 1 | ZLXV-1 | 1980 | 14.74 | PV190946 | African horse sickness virus VP1 (horse, South Africa, AHI44045) | dsRNA (segmented) | *Sedoreoviridae* | *Orbivirus* | 0E+00 | 63.19 | 1 | 1 |
| Zaloxo virus 2 | ZLXV-2 | 500 | 1.28 | PV190956 | Totiviridae sp. (tick, China, WAK77261) | dsRNA (linear) | *Totiviridae* | unclassified | 4E-59 | 64.78 | 0 | 2 |
| Zaloxo virus 3 | ZLXV-3 | 561 | 1.45 | PV190957 | Virus sp. Rep (unspecified, USA, QSM07494) | ssDNA (circular) | *Circovirdae* | unclassified | 1E-113 | 98.76 | 1 | 0 |
| Zaloxo virus 4 | ZLXV-4 | 542 | 4.68 | PV190958 | Sorex Araneus polyomavirus 1 VP1 (shrew, Germany, ASU50401) | dsDNA (circular) | *Polyomaviridae* | *Alphapolyomavirus* | 1E-95 | 74.59 | 6 | 3 |
| Zaloxo virus 4 | ZLXV-4 | 525 | 1.42 | PV190959 | Sorex coronatus polyomavirus 1 VP2 (shrew, Germany, YP_010084743) | dsDNA (circular) | *Polyomaviridae* | *Alphapolyomavirus* | 2E-75 | 64.37 | 5 | 2 |

^1^Average sequence coverage; ^2^ GenBank accession number of viral sequence from this study. Accession number for genes on all 10 ZLXV-1 segments are PV190946-PV190955; ^3^Closest match, genome composition, family, genus, E-value, and percent identity (amino acid, to the closest match) from querying the deduced amino acid sequence of each contig against the NCBI nonredundant (nr) protein database using blastp; and ^4^Number of tick salivary glands (SG) and blood meals (BM) in which each virus was identified, out of 6 SG and 4 BM tested.

**
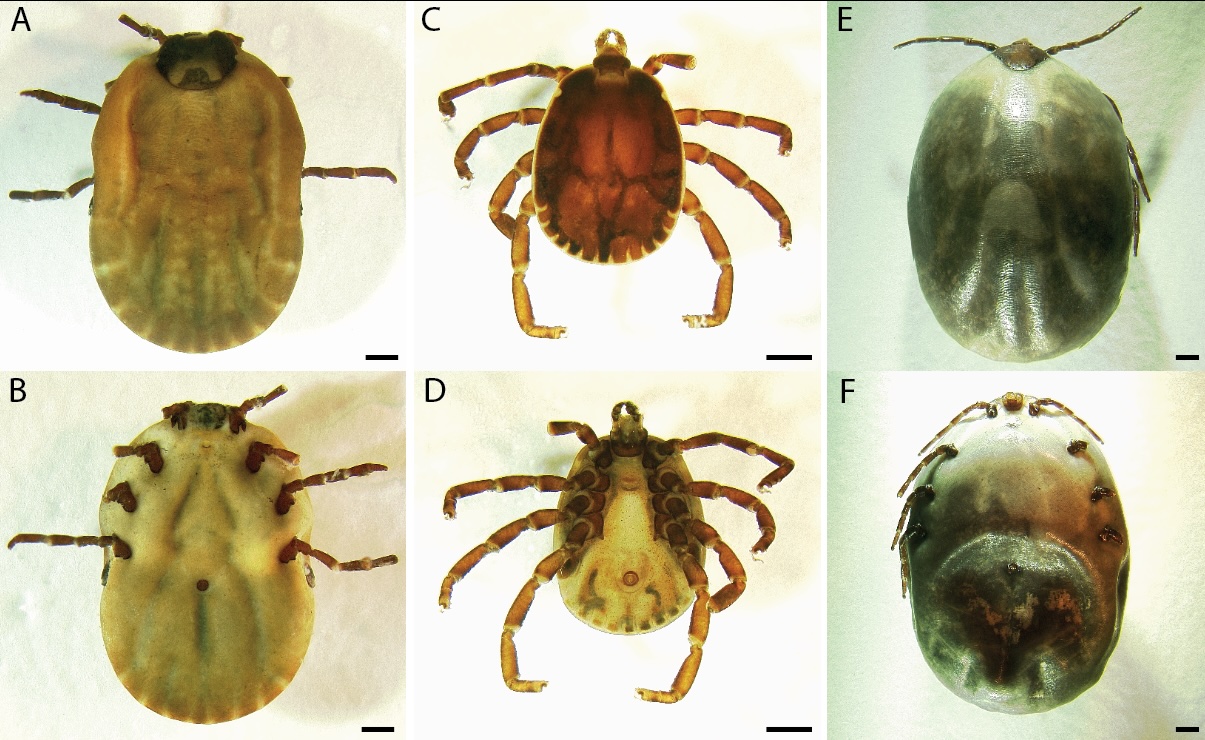
**

**Figure 1S.** Dorsal (top row) and ventral (bottom row) images of ticks recovered from elephants residing in Kafue National Park, Zambia. A, B: *Rhipicephalus maculatus* (engorged); C, D: *Amblyomma tholloni* (unengorged); E, F: *Amblyomma tholloni* (engorged). Scale bars = 1 mm.
